# Of Plants and Men

**DOI:** 10.1002/cfg.77

**Published:** 2001-04


**Comparative and Functional Genomics**
*Comp Funct Genom* 2001; **2**: 59.
**DOI**: 10.1002/cfg.77

**Editorial**

### Of plants and men

Much attention is currently focused on the scientific, ethical, and political implications of the draft sequence of the human genome. In line with this, the next issue of *CFG* will carry pieces from two researchers in the field of human genetics/genomics on the impact that the draft sequence will have on their research. Andrew Simpson (Ludwig Institute, Sao Paulo, Brazil) will review the importance of ESTs after the release of both the draft sequence and the first estimate of the number of human genes based on hard data. There will also be an interview with Duncan Campbell on the changing role of the UK Medical Research Council's Human Genome Mapping Project Resource Centre in Hinxton, now that the human genome sequence has been released.

In contrast to the rather acrimonious relationships between some of the principal centres involved in sequencing the human genome, the *Arabidopsis* Genome Project was a highly collaborative worldwide effort and the subsequent phase of functional genomics looks like continuing in the same vein. In this issue of *CFG*, we have *Arabidopsis* as our 'featured organism'. The article includes comments from Pam Green (*Arabidopsis* Functional Genomics Consortium and Michigan University, USA) and Sean May (Nottingham *Arabidopsis* Stock Centre, UK). We also have an interview with Mike Bevan (John Innes Centre, UK), who led the European sequencing effort. A number of funding bodies across the world are sponsoring the functional genomic analysis of the humble thale cress. The US has its 2010 program to determine the function of all *Arabidopsis* genes by that date. The European Union has established EXOTIC (EXOn Trapping Insert Consortium) and REGIA (REgulatory Gene Initiative in *Arabidopsis*) to investigate *Arabidopsis* gene expression patterns and transcription factors, respectively. In the UK, the BBSRC (Biotechnology & Biological Sciences Research Council) is sponsoring *Arabidopsis* functional analysis through its 'Investigating Gene Function' initiative.

In all this excitement about multicellular organisms, we should not forget my favourite beasts – the yeasts. The next issue of *CFG* will feature the fission yeast *Schizosaccharomyces pombe*, one of the major models for the study of the eukaryotic cell cycle, whose genome has just been sequenced by a Sanger Centre/EU consortium. The current issue of *CFG* includes an article on the complete sequence of the mitochondrial genome of *Yarrowia lipolytica* – the hydrocarbon-utilising yeast that has been commercially exploited in the production of citric acid, as well as being an important model for the study of protein secretion.

**Steve Oliver**

Copyright © 2001 John Wiley & Sons, Ltd.

